# On the Detection of Arsenic, in Answer to Mr. Prideaux

**Published:** 1819-02

**Authors:** J. Hume


*For the London Medical and Physical Journal.*

### *On the Detection of Arsenic, in Answer to Mr. Prideaux;* by J. Hume, Esq.

ALTHOUGH the chief tendency of some of my late letters on the detection of arsenic has been to defend what I have long considered to be unassailable, I have never neglected, as often as an occasion presented itself, to blend controversy with instruction. The position I have assumed, and now fixed, is this—"that nothing is more readily and securely detected than arsenic; and that its re-production into the state of *white* oxide can never be liable to deception." I may with the same confidence add, that, to prefer any process for obtaining the poison in its *metallic* form, when the quantity is small, and probably only suspected to exist, argues either great poverty of experience, or excessive and unreasonable obstinacy in such advocates.

Mr. Prideaux has essentially misconceived nearly the whole of my letter in your Journal for October; the most material parts of it are either garbled or misquoted. All the progress of the experiments, after the words "this proof alone," until the evidence before the coroner's jury, has been omitted; nor has he noticed the advantages that are derived from the very insoluble quality of white arsenic, which is so contrary to that of the only phosphates that could interfere. Let Mr. Prideaux again consider my letter, particularly the experiments upon the contents of the coffee-cup; he will, at least, see that no acidulated phosphate of soda could be in my way.

The first paragraph of Mr. Prideaux's last letter seems to acknowledge all I could wish; so that, notwithstanding the other parts, I no longer deem him an opponent. My time at present is too much occupied to allow me to enter fully upon some of his objections; all of which are already obviated, if he will but re-consider my letters. I had, indeed, nearly determined to let the matter rest here, but that one circumstance he has mentioned calls for an early observation: it is where my words are mis-quoted in such a way that it would appear, from one proof or experiment only, I took upon myself the whole responsibility of proceeding to an inquest. At page 470, line 12, Mr. Prideaux writes *my* instead of *our*; whereas, if the context be taken in, your readers will find, that "Mr. Holdsworth and I then agreed to the propriety of *dissection*, and that the contents of the stomach should also be obtained to *aid* the enquiry." On numerous occasions, a coroner's inquest implicates no person's character: indeed, it is always a court of inquiry, and the result is often an unoffending and simple verdict,—such as "Died by the visitation of God;" "Found drowned;" "Died by suffocation," &c.

I could wish Mr. Prideaux had been silent about the Launceston trial, especially on the "three points of evidence." I am very unwilling to press the subject, and again to touch upon the evidence that was displayed on that occasion. The anecdote which I quoted in a former letter proves that all the reasonings of an incompetent witness are mere empty vapours; they are of no value as testimony. In this sentence (I trust I shall be fully understood) not the slightest allusion is meant to Mr. Prideaux, whose abilities and character I have every inducement to respect: he writes with temper, and it shall be my duty to follow his example.

*Long Acre; Dec.* 14, 1818.

